# Balancing selection and trans-specific polymorphisms

**DOI:** 10.1186/s13059-017-1365-1

**Published:** 2017-12-12

**Authors:** Baosheng Wang, Thomas Mitchell-Olds

**Affiliations:** Center for Genomic and Computational Biology, Department of Biology, Duke University, Box 90338, Durham, NC 27708 USA

## Abstract

Balancing selection maintains variation for evolution. A recent study investigated the extent of balancing selection in two Brassicaceae species and highlighted its importance for adaptation.

## Introduction

Populations of plants and animals show extensive variation for traits and for the nucleotide polymorphisms that underlie phenotypic differences. The evolutionary factors that influence this variation include neutral genetic drift, weakly deleterious mutations with short persistence times in populations, and advantageous alleles that are increasing in frequency. In addition, balancing selection causes elevated levels of nucleotide polymorphism that exceeds neutral levels, actively maintaining multiple alleles in a gene pool at higher-than-expected frequencies. In some cases, balancing selection may be identified by trans-specific polymorphisms (TSPs).

In their recent study, Guo and colleagues [1] investigated the importance of balancing selection in maintaining genetic variation and promoting local adaptation in two Brassicaceae species, *Arabidopsis thaliana* and its close relative *Capsella rubella*, which diverged about 8 million years ago.

## Balancing selection in plants and animals

The processes that maintain balanced polymorphisms include negative-frequency-dependent selection (where rare alleles are favored), temporal or spatial variation in selection, interactions of genotype effects with sex or age, and, occasionally, overdominance (single locus heterozygote advantage) [2]. These processes are well understood for genes of large effect, but the relative importance of balancing selection on complex traits remains unclear.

Leffler et al. [3] conducted an early genome-wide scan for long-term balancing selection by looking for TSPs between humans and chimpanzees, identifying a large number at immune loci such as the major histocompatibility complex (MHC) genes and in blood group genes, as well as several candidate targets outside of these classic examples. That study suggested that balancing selection has shaped genetic variation in the human genome and could maintain polymorphisms for millions of years. Taking advantage of existing whole-genome sequences for *A. thaliana* and related species [4, 5], Guo and colleagues [1] obtained around 4.9 million single nucleotide polymorphisms (SNPs) in 80 *A. thaliana* accessions and around 2.1 million SNPs among 22 *C. rubella* accessions. By conducting a genome-wide scan for TSPs, the authors detected five candidate genes under balancing selection, and further ecological modeling suggests possible adaptation to divergent habitats in *A. thaliana*.

## Investigating balancing selection in Brassicaceae

Guo and colleagues [1] compared whole-genome variation of the two species to identify TSPs. Owing to the large number of genes compared, they used a series of stringent filtering steps to reduce false positives. (Such false positives, in which TSPs were generated by other evolutionary processes rather than by balancing selection, would mislead our understanding of the extent and importance of balancing selection in genome evolution.) To avoid misinterpreting variation among gene copies (paralogs) as polymorphisms at a single locus, they focused on 16,014 conserved, orthologous, single-copy gene pairs, which contained 1.1 and 0.45 million bi-allelic SNPs in *A. thaliana* and *C. rubella*, respectively. Among these polymorphic sites, 8535 SNPs showed pairs of shared SNPs (shSNP) between species. Because alignments in coding regions are more reliable than those in non-coding sequences, the authors retained only about one-third of the high-quality shSNPs found in coding regions, affecting 433 genes.

These shSNPs might reflect neutral evolutionary processes, such as incomplete lineage sorting of ancestral polymorphisms, or recurrent mutation instead of balancing selection. To understand the potential for neutral factors to maintain shared polymorphisms, Guo and colleagues [1] inferred the demographic history of *A. thaliana* and *C. rubella* by using coalescent simulations. Historical reductions in population size (bottlenecks) were detected in both species following divergence from their common ancestor. In addition, these analyses indicate that ancient gene flow occurred between ancestors of these two species. On the basis of neutral coalescent theory and estimated demographic parameters, the probability of incomplete lineage sorting (i.e., that two *A. thaliana* and *C. rubella* alleles have not coalesced in the interval since speciation) is in the order of 10^–9^. This implies that < 1 shSNP would be retained in aligned genomic regions under genetic drift alone. This estimated probability still applies with selfing and population structure within species, and is unlikely to be influenced by ancestral gene flow. Therefore, the existence of shSNPs cannot be explained by genetic drift alone, and they are probably maintained by balancing selection.

**Fig. 1:**
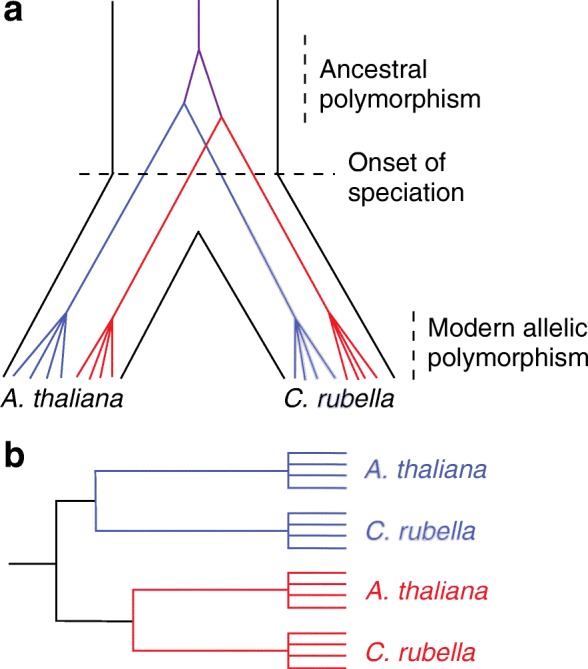
Origin and identification of trans-specific polymorphisms at a single-copy locus. **a** Time runs from top to bottom, and variation within a species is enclosed by flanking black lines. Before the onset of speciation, two alleles segregate within the ancestral species (*purple lines*), and each copy gives rise to a pair of descendant alleles (two *blue* or two *red*). As speciation proceeds, these descendant alleles are inherited in the daughter species, and finally modern allelic polymorphism exists among individuals. (As is typical in coalescent analyses, only lineages that are represented in our modern sample are shown.) **b** An allele phylogeny showing the relationships of modern, sequenced alleles at one single-copy gene. In the *blue* clade, *Arabidopsis thaliana* alleles are more closely related to blue alleles in *Capsella rubella* than to *red* alleles in *A. thaliana* because of trans-specific polymorphism. Figure adapted from Wu et al. [1]

Under neutrality, haplotypes carrying the ancestral polymorphism may be broken up as the result of recombination, and it is difficult to identify non-recombinant alleles for species that diverged long ago. By contrast, balancing selection can suppress recombination around selected sites, and short ancestral segments that harbor multiple linked variants might persist until all lineages coalesce to their common ancestor. In this context, ancient balanced polymorphisms may be clustered by allelic type rather than by species (Fig. 1a and b), an indication of balancing selection. On the basis of a recombination rate of 3.6 cM/Mb for *A. thaliana* and *C. rubella*, Guo and colleagues [1] estimate that old, neutrally evolving segments would be only several base pairs in length. Therefore, they scanned 100-bp sliding windows across the 433 identified candidate genes to find sequence regions that are clustered by alleles rather than species (Fig. 1b). To reduce the chance of false positives, a number of filtering steps were applied.

Guo and colleagues [1] then identified haplotypes from five genes as candidate TSPs under long-term balancing selection. These five genes are single copy in both species, and simulation studies confirmed that this pattern would be very unlikely under neutral evolution, suggesting that these five TSPs are maintained by balancing selection. Balancing selection was also supported by high nucleotide diversity and intermediate frequency polymorphism in these regions, as expected for ancient balanced polymorphisms. The five candidate genes are associated with different biological and biochemical processes, including response to biotic and abiotic stress.

Finally, Guo and colleagues [1] examined the roles of these five candidate genes in adaptation to divergent habitats. They focused on *A. thaliana* because of the extensive information on the genetic, geographical, and ecological variation in this species. To avoid confounding with historical genetic divergence, they considered four genes that were independent of population history and that correlated with ecological divergence, suggesting local adaptation. Environmental niche modeling confirmed that two allelic groups of the four genes occupied significantly different niches, and expression analyses detected different expression levels between haplotype groups in one of the four genes. Taken together, these results indicate that genes under balancing selection may have contributed to adaptation in *A. thaliana*.

## Perspectives

While previous research has revealed a handful of genes that are under balancing selection in plants [6, 7], few studies have analyzed the footprints of long-term balancing selection on a genome-wide scale in closely related species pairs [1, 8]. Given the stringent filtering criteria used, it is not surprising that only five candidate genes were identified. These filtering steps are necessary to avoid false positives, although some true TSPs may have been filtered out. In addition, non-coding regions that were excluded from data analyses might contain regulatory regions that are under balancing selection; such regions may be identifiable as long-read sequencing technologies become more cost effective.

Additional approaches are feasible for future work. For example, biological mechanisms may be revealed if nearly significant genes are enriched in particular pathways, as demonstrated by similar approaches in genome-wide association studies [9] and population genetics [10]. In addition, larger numbers of TSPs may be found when comparing more closely related species pairs, provided that neutral lineage sorting is largely complete. Finally, physiological or field experiments can provide more information on the molecular and ecological mechanisms that contribute to balancing selection and TSPs.

## Abbreviations

TSP: Trans-specific polymorphism
shSNP: Shared SNP
SNP: Single nucleotide polymorphism
